# P-1350. Global mortality in Acinetobacter baumannii-caused ventilator associated pneumonia treated with colistin monotherapy in an intensive care unit

**DOI:** 10.1093/ofid/ofaf695.1538

**Published:** 2026-01-11

**Authors:** Sandra V Aronson, Lorena Abusamra, Victoria Pinto, Ivan Assof, Maria Jose Rolon, Laura Errecalde, Sandra Cogut, Cecilia Vargas Ramos, Franz Arancibia Morales, Ernesto Abecian, Roberto Villa, Alicia Sisto

**Affiliations:** Hospital General de Agudos Juan A Fernandez, Buenos Aires, Ciudad Autonoma de Buenos Aires, Argentina; Hospital General de Agudos Juan A Fernandez, Buenos Aires, Ciudad Autonoma de Buenos Aires, Argentina; Hospital General de Agudos Juan A Fernandez, Buenos Aires, Ciudad Autonoma de Buenos Aires, Argentina; Hospital General de Agudos Juan A Fernandez, Buenos Aires, Ciudad Autonoma de Buenos Aires, Argentina; Hospital Dr. Juan A. Fernández, Buenos Aires, Ciudad Autonoma de Buenos Aires, Argentina; Hospital General de Agudos Juan A Fernandez, Buenos Aires, Ciudad Autonoma de Buenos Aires, Argentina; Hospital General de Agudos Juan A Fernandez, Buenos Aires, Ciudad Autonoma de Buenos Aires, Argentina; Hospital General de Agudos Juan A Fernandez, Buenos Aires, Ciudad Autonoma de Buenos Aires, Argentina; Hospital General de Agudos Juan A Fernandez, Buenos Aires, Ciudad Autonoma de Buenos Aires, Argentina; Hospital General de Agudos Juan A Fernandez, Buenos Aires, Ciudad Autonoma de Buenos Aires, Argentina; Hospital General de Agudos Juan A Fernandez, Buenos Aires, Ciudad Autonoma de Buenos Aires, Argentina; Hospital General de Agudos Juan A Fernandez, Buenos Aires, Ciudad Autonoma de Buenos Aires, Argentina

## Abstract

**Background:**

Ventilator-associated pneumonia (VAP) has been considered as a healthcare-associated infection with high mortality. *Acinetobacter baumannii* is one of the most frequently isolated pathogens in hospitals worldwide, especially in critically ill patients who needed invasive mechanical ventilation. *Acinetobacter* ventilator associated pneumonia (AB-VAP) is associated with high case-fatality rates, reaching 70% in some studies, with controversy still existing on the ideal antimicrobial treatment.

**Methods:**

We performed a descriptive, retrospective study. To meet the inclusion criteria, patients had to be adults admitted to the intensive care unit with VAP diagnosis treated with colistin monotherapy and isolation of *Acinetobacter baumannii* in lower respiratory tract samples as the sole pathogen between December 2016 and December 2023. We evaluated demographic variables, time between ICU admission and the clinical event, case-fatality rate and antimicrobial susceptibility profile. We calculated with RStudio the average (and standard deviation) or median (and interquartile range values) for quantitative variables and frequency for qualitative ones. The antimicrobial choice was influenced by local susceptibility patterns and the economic resources of our hospital.

**Results:**

Data from 126 patients that met the inclusion criteria was analyzed. The mean age was 55.5 years (SD:16 years) and 68% were men. Median time between he ICU admission and VAP diagnosis was 14 days (percentiles 25-75%: 8-27 days). Median Charlson Comorbidity Index value was 3, with an interquartile range of 1 - 5.

Case fatality rate at 14 days after the diagnosis was 30,4% (IC95%: 23-39), reaching 36,8% at 28 days (IC95%: 28,8-45,5).All microbiological isolates, except one, were susceptible to colistin and none to carbapenems

**Conclusion:**

Although mortality in patients with AB-VAP remains high in our hospital, it does not reach the values described in the literature, even when treated with non-beta-lactam monotherapy. We also observed that the highest mortality is concentrated in the first 14 days after the infection diagnosis. Larger multicenter studies are needed to confirm our findings.

**Disclosures:**

All Authors: No reported disclosures

